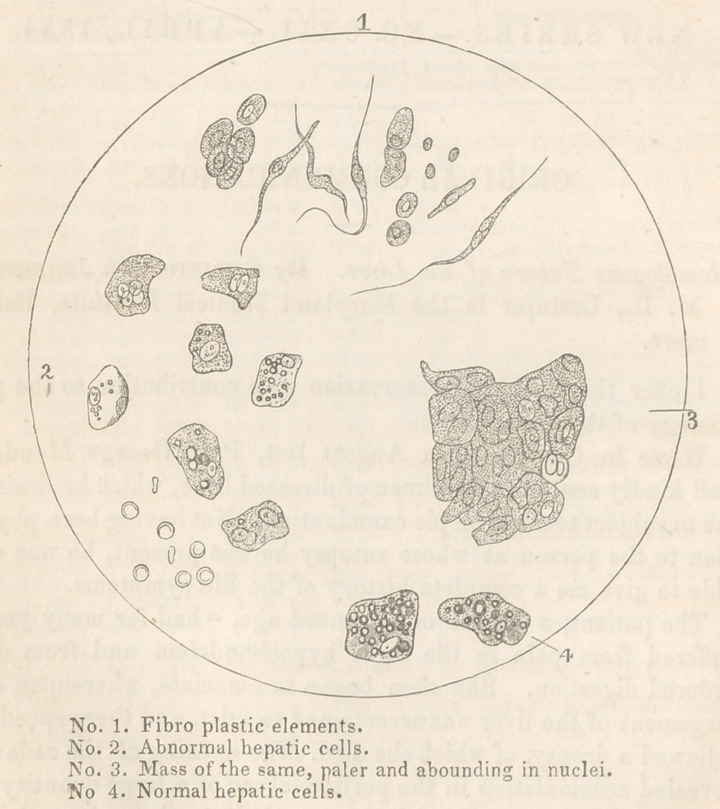# Homologous Tubera of the Liver

**Published:** 1854-04

**Authors:** Christopher Johnston

**Affiliations:** Lecturer in the Maryland Medical Institute, Baltimore


### MEDICAL EXAMINER.

NEW SERIES.—NO. CXII.—APRIL, 1854.

---

### ORIGINAL COMMUNICATIONS.

---

*Homologous Tubera of the Liver.* By CHRISTOPHER JOHNSTON, M. D., Lecturer in the Maryland Medical Institute, Baltimore.

I offer the following observation as a contribution to the pathology of the liver.

While in Cincinnati, in August last, Prof. George Mendenhall kindly sent me a specimen of diseased liver, which he desired me to subject to microscopic examination. Not having been physician to the person at whose autopsy he was present, he was unable to give me a complete history of the life symptoms.

The patient, a female of advanced age, "had for many years suffered from pain in the right hypochondrium and from disordered digestion. She then began to emaciate, whereupon enlargement of the liver was ascertained to exist, and there speedily followed a dropsy, of which she died. The opening of the cadaver revealed accumulation in the peritoneal sac of a large quantity of water, and a liver of much increased dimensions, irregularly bosselated, yellowish upon the projecting points, but fading gradually into the hepatic tissue at the base of the elevation, which measured from a half inch to several inches in diameter. No other lesion could be discovered."

The general appearance of the tumors corresponded very nearly to the description given in Hope's Pathological Anatomy (p. 133) of "Cancerous tubera," but the microscope detected *no heterologous elements*, but homologous ones only, and in a relation not hitherto set forth.

The tumors were of two sorts, the *hard* and the *soft*, the former being composed principally of fibro-plastic matter; the latter consisting of altered *hepatic cells* mingled with the fibro-plastic elements, particularly near the tissue as yet unchanged.

No. 1. Fibro plastic elements.

No. 2. Abnormal hepatic cells.

No. 3. Mass of the same, paler and abounding in nuclei.

No 4. Normal hepatic cells.

The change in the hepatic cells occurs in this wise: they first become pale, and lose their fatty globules, while at the same time the nucleus in each increases in size, and undergoes bipartite segmentation. A new brood of cells starts into existence, but in every new generation the capsule presents successively a smaller diameter, until at length it ceases altogether; but the nucleus multiplies itself indefinitely, so that in the centre of the tumor nothing is to be found save a mass of free nuclei.

A tumor may be compound, that is, may result from the fusion of two or more simple ones.

In proportion as this process advances, the affected part, or the tumor, softens in the middle, (and not on the border where the fibro-plastic element is most abundant,) extravasation of blood takes place, and the coagulum is diffuse.

This diseased condition must react in two ways upon the constitution.

The quantity and quality of *sugar* produced in the liver must be affected in the ratio of the modification taking place in the essential hepatic glandular element; and the bulk of the organ and its weight must make themselves felt as obstacles to the circulation of blood, particularly in the venous system. In other words, the respiratory and assimilative functions suffer first, next the digestion, by reason of the impediment to the circulation in the portal vein, and then dropsy has an easy explanation.

*Baltimore, Jan.* 19, 1854.

## Figures and Tables

**Figure f1:**